# Non-uniform recovery of left ventricular transmural mechanics in ST-segment elevation myocardial infarction

**DOI:** 10.1186/1476-7120-8-31

**Published:** 2010-08-03

**Authors:** Giuseppe Caracciolo, Mackram F Eleid, Haruhiko Abe, Nisha Bhatia, F David Fortuin, Susan Wilansky, Scipione Carerj, Partho P Sengupta

**Affiliations:** 1 Division of Cardiovascular Diseases, Mayo Clinic Arizona 13400 East Shea Blvd, Scottsdale, AZ 85259 USA

## Abstract

**Background:**

After a transient ischemic episode, the subendocardial region is more severely injured than outer subepicardial layers and may regain a proportionately greater degree of mechanical function in the longitudinal direction. We sought to explore left ventricular (LV) transmural mechanics in patients with ST-segment elevation myocardial infarction (STEMI) for determining the mechanism underlying recovery of global LV function after primary percutaneous coronary intervention (PCI).

**Methods:**

A total of 42 patients (62 ± 11 years old, 71% male) with a first STEMI underwent serial assessments of LV longitudinal, circumferential and radial strains (LS, CS and RS) by selective tracking of subendocardial and subepicardial regions within 48 hours and a median of 5 months after PCI. LV mechanical parameters were compared with sixteen age and gender matched normal controls.

**Results:**

In comparison with controls, endocardial and epicardial LS were markedly attenuated at 48 hours following PCI (P < 0.001). An improvement in LV ejection fraction (EF > 5%) following PCI was seen in 24 (57%) patients and was associated with improvement in endocardial and epicardial LS (P < 0.001 and P = 0.003, respectively) and endocardial CS (P = 0.01). Radial strain and wall motion score index, however, remained persistently abnormal. The change in endocardial LS (OR 1.2, 95% CI 1.03 to 1.42, P = 0.01) and the change in epicardial LS (OR 1.2, 95% 1.03 to 1.46, P = 0.02) were significantly associated with the improvement in LVEF, independent of the location of STEMI and the presence of underlying multivessel disease.

**Conclusions:**

In patients with STEMI treated by PCI, the recovery of LV subendocardial shortening strain seen in the longitudinal direction underlies the improvement in LV global function despite persistent abnormalities in radial mechanics and wall motion score index.

## Background

Left ventricular (LV) structure and function exhibits substantial transmural heterogeneity. Myofiber orientation changes gradually from a right-handed helix in the subendocardium to a left-handed helix in the subepicardium [1]. Besides redistributing stresses and strains uniformly along the transmural layers, the changing helical orientation contributes to shear deformation in which myocardial fibers slide over each other [1-4]. The inward shearing of myofibers accounts for the greater radial thickening strains (> 40%) seen over the endocardium despite relatively small myocyte contraction (about 15%) [5].

Contractile dysfunction after ischemia and reperfusion in vitro is associated with a significant transmural gradient of dysfunction between epicardial and endocardial layers [6]. The serial changes in epicardial and endocardial mechanics that result in global improvement in LV systolic function following reperfusion in the intact heart, however, are not fully understood. The severity of ischemia during restriction of arterial inflow is greater in the subendocardium compared with the subepicardium [7]. Accordingly, it is conceivable that transient ischemia and reperfusion may result in a proportionately greater degree of mechanical function being restored in the longitudinal direction due to greater recovery of subendocardial function.

In this investigation we explored the longitudinal, circumferential and radial mechanics of the LV in patients with ST-segment elevation myocardial infarction (STEMI) for determining the selective contribution of subendocardial and subepicardial region to the recovery of global LV function after primary percutaneous coronary intervention (PCI). We hypothesized that there is preferential recovery of subendocardial longitudinal shortening in STEMI after primary percutaneous coronary intervention (PCI).

## Methods

The Mayo Clinic Institutional Review Board approved the study. We retrospectively studied and enrolled 42 patients (62 ± 11 years old, 71% male) with their first STEMI referred to the catheterization laboratory at our institution for emergency PCI between January 1, 2005 and December 31, 2008. Patients presenting within six hours of the onset of symptoms suggesting an acute myocardial infarction, associated with ≥ 0.2 mV (2 mm) ST elevation in ≥ 2 contiguous anterior leads or limb leads with a summed ST elevation ≥ 1.0 mV (10 mm) in all leads on the presenting electrocardiogram, and in whom primary PCI was planned, were eligible for this study. The exclusion criteria were fibrinolytic therapy, previous infarct history, atrial fibrillation, significant valvular heart disease, life-limiting non-cardiac disease, previous history of chronic congestive heart failure. Serial clinical data including demographics, co-morbid conditions, and laboratory results were recorded for each individual. All patients underwent a comprehensive echocardiographic examination within 48 hours and during follow up (median 5 months) after the primary PCI. LV mechanical parameters were compared with 16 subjects served as controls with similar age and gender to the study population (63 ± 11 years, 9 males) with normal echocardiograms. Control group risk factors included hypertension in 6, dyslipidemia in 5, diabetes in 1 and family history of cardiovascular diseases in 2, but none had coronary artery disease or known structural heart disease.

### Echocardiography

Echocardiographic studies were performed on commercially available ultrasound equipment (Acuson Sequoia, Siemens Medical, Mountain View, CA and Vivid-7, GE Healthcare, Milwaukee, WI) according to the standard method recommended by the American Society of Echocardiography. The LV volumes and ejection fraction (EF) were obtained by the modified biplane Simpson's method from the apical 4- and 2-chamber views. The LV wall was divided into 16 segments and the wall motion of each segment was visually evaluated and scored with the following scale: 1 = normal; 2 = hypokinesis; 3 = akinesis; 4 = dyskinesis; and 5 = aneurysmal. The average of the score of evaluated segments served as the WMSI. The mitral early diastolic flow (E) velocity and late diastolic flow (A) velocity were measured and the E/A ratio calculated. The deceleration time of the mitral E wave was also measured and Doppler tissue imaging was obtained from the apical four-chamber view. The digital images were obtained at optimal frame rates (≥ 30 frames per second). Images were stored in digital cineloop format (Prosolv Cardiovascular solutions, Indianapolis, IN) for offline analysis by vendor customized 2-D Cardiac Performance Analysis software (2D CPA, TomTec multimodality imaging solution, Munich, Germany). 2D CPA is a speckle tracking based analysis tool that can analyze 2D data from various ultrasound machines and is an extension of velocity vector imaging software that has been previously validated with sonomicrometry [8,9] and magnetic resonance imaging [10,11]. 2D CPA, similar to velocity vector imaging, determines myocardial motion from a user-defined tracing along the endocardial border. Both users defined endocardial and automated subepicardial borders are traced throughout one cardiac cycle by successive application of a series of tracking steps. From this motion, the myocardial velocity, longitudinal and radial strain are calculated for both endocardial and subepicardial regions along the trace. Longitudinal systolic strain from endocardial and subepicardial regions respectively was obtained from 6 segments and from lateral and septal wall segments in apical 4-chamber views. Circumferential strain and radial strain were obtained from 6 segments in short-axis views of the LV at the level of papillary muscle. Assessment of the LV strain was regarded as suboptimal when either: 1) speckle tracking could not be obtained for at least 4 of the 6 myocardial segments in apical 4-chamber or short-axis views; or 2) a theoretically unacceptable value or values were obtained. Offline analyses were independently performed by one observer who was not involved in image acquisition nor had knowledge of other echocardiographic measures of LV function. Echocardiographic indices were measured as per the recommendations of American Society of Echocardiography [12]. Serial changes in global strains and WMSI were compared for predicting an improvement of LV ejection fraction (EF > 5%) on follow-up.

### Statistical Analysis

All continuous data were reported as mean ± SD, and categorical data, as percentage. Chi-square and the unpaired Student t test were used for comparisons between two groups for categorical and continuous variables, respectively. A P value less than 0.05 was considered statistically significant. Pearson's correlation coefficient was used to reveal relations between two continuous variables. Univariate logistic regression analysis was used to compare clinical and echocardiographic variables with improvement in LVEF during follow up using commercially available software (MedCalc 11.2 software MariaKerke, Belgium). Inter- and intra-observer variability was calculated as the absolute difference of the corresponding pair of repeated measurements in percent of their mean in each patient and then averaged for 18 randomly selected patients. To evaluate intra observer agreement among two readers of WMSI, the mean kappa and its 95% confidence interval was calculated. The kappa coefficient of agreement was graded as follows: 0 to 0.2 = poor to slight; 0.21 to 0.4 = fair; 0.41 to 0.6 = moderate; 0.61 to 0.8 = substantial; and 0.81 to 1.0 = nearly perfect.

## Results

The clinical characteristics of STEMI patients with and without improvement in LVEF at baseline are summarized in Table 1. On analysis of coronary risk factors, there was a trend towards less hypertension, hyperlipidemia and diabetes in patients showing improvement in LVEF. However, there was no difference between the two groups with respect to baseline demographic data, infract location, infract size as suggested by peak creatine kinase MB and peak troponin values, door to balloon time, extent of coronary artery disease, Killip Class, TIMI score, or post-procedural complications. There were no also no differences in utilization of medications during follow-up (Table 2).

**Table 1:** Baseline Characteristics Data

	No improvement	Improvement	
	(n = 18)	(n = 24)	P value
Age (years)	64.0 ± 8.7	61.7 ± 12.8	0.48
Sex (female)	6 (33%)	6 (25%)	0.80
Body Mass Index (kg/m^2^)	29.6 ± 5.3	28.7 ± 6.3	0.62
Systolic Blood Pressure (mmHg)	130.8 ± 18.9	140.3 ± 24.2	0.16
Diastolic Blood Pressure (mmHg)	73 ± 13.7	83.8 ± 15.5	0.02
Heart Rate (beat/min)	77 ± 13	82.6 ± 23.5	0.37
Door to balloon time (minutes)	71 ± 42	69 ± 31.6	0.85
Hospitalization (days)	5.4 ± 1.9	6.6 ± 4.3	0.24
Peak Creatine kinase MB (U/L)	161.4 ± 148.0	183.0 ± 249.2	0.72
Peak Troponin (ng/mL)	6.4 ± 5.6	5.5 ± 6.8	0.64
Risk factors			
Hypertension	12 (67%)	8 (33%)	0.06
Diabetes	4 (22%)	1 (4%)	0.19
Dyslipidemia	12 (67%)	8 (33%)	0.06
Killip Class			
I	13 (72%)	18 (75%)	0.87
II	1 (6%)	3 (17%)	0.81
IV	4 (22%)	3 (17%)	0.67
Anterior wall Myocardial Infarction	10 (56%)	9 (47%)	0.39
Disease extent			
1 Vessel Disease	8 (44%)	14 (59%)	0.56
2 Vessel Disease	5 (28%)	5 (21%)	0.87
3 Vessel Disease	5 (28%)	5 (21%)	0.87
IABP	4 (22%)	3 (17%)	0.67
Number of stents			
1	11 (61%)	12 (67%)	0.68
2	4 (22%)	8 (44%)	0.65
3	1 (6%)	4 (22%)	0.53
4	2 (11%)	-	0.34
TIMI Score pre PCI			
0	13 (72%)	18 (75%)	0.87
1	1(6%)	-	0.88
2	4 (22%)	5 (21%)	0.78
3	-	1 (4%)	0.88
TIMI Score post PCI			
2	1(6%)	2 (8%)	0.79
3	17 (94%)	22 (92%)	0.79
Complications			
A-V Block	1 (6%)	3 (17%)	0.81
Ventricular Arrhythmia	4 (22%)	4 (22%)	0.95
Atrial Arrhythmia	3 (17%)	7 (39%)	0.56

**Table 2:** Utilization of Medications after PCI

	No improvement	Improvement	
	(n = 18)	(n = 24)	P value
Aspirin	18 (100%)	23 (96%)	0.88
Clopidogrel	17 (94%)	22 (92%)	0.79
Nitrates	1 (6%)	-	0.88
PRN NTG	12 (67%)	11 (46%)	0.30
Coumadin	3 (17%)	5 (21%)	0.95
Beta-blocker	16 (89%)	24 (100%)	0.34
ACE inhibitor or ARB	16 (89%)	19 (80%)	0.67
Statins	17 (94%)	21 (88%)	0.81
Number of patients requiring changes in drug dosage at follow up			
Beta-blocker	5 (28%)	9 (47%)	0.74
ACE inhibitor or ARB	5 (28%)	8 (33%)	0.96
Statins	3 (17%)	6 (25%)	0.78

Table 3 summarizes the echocardiographic data of both groups of patients ≤ 48 hours after PCI and during follow up. There were no significant group differences in WMSI, LV end-systolic and end-diastolic volume, cardiac index, thickness, volume index, A and E velocity, E-A ratio, deceleration time, E/e', RA pressure, RV systolic pressure. LVEF (46.0 ± 10.7 vs. 53.4 ± 14.1, P < 0.05) was significantly improved during follow up in a group of patients.

**Table 3:** Dimensional Echocardiographic Data

	Post PCI		Follow-up	
	No Improvement	Improvement	No Improvement	Improvement
Wall Motion Score Index	1.5 ± 0.4	1.5 ± 0.3	1.5 ± 0.3	1.3 ± 0.4
Ejection Fraction (%)	49.9 ± 11.1	46.0 ± 10.7	51.9 ± 13.5	53.4 ± 14.1§
LVEDV (mL)	65.8 ± 23.2	53.4 ± 15.0	72.8 ± 26.4	62.9 ± 23.9
LVESV (mL)	32.6 ± 12.5	29.5 ± 12.7	37.2 ± 20.7	29.5 ± 17.3
Cardiac Index (l/min/m2)	2.7 ± 0.4	2.9 ± 0.8	2.6 ± 0.6	2.7 ± 0.5
LV Mass index (g/m^2^)	121.3 ± 31.0	103.2 ± 30.4	113.1 ± 33.6	92.6 ± 24.2†
LA Volume Index (4C-2C) (cc/m^2^)	37.3 ± 8.5	32.5 ± 13.6	37.4 ± 18.2	31.3 ± 7.9
A velocity (m/sec)	0.7 ± 0.2	0.7 ± 0.2	0.7 ± 0.2	0.7 ± 0.2
E velocity (m/sec)	10.6 ± 39.2	0.8 ± 0.1	0.7 ± 0.2	0.8 ± 0.2
E-A ratio	1.2 ± 0.7	1.2 ± 0.4	1.0 ± 0.3	4.2 ± 13.7
Deceleration time (m/sec)	181.7 ± 66.8	191.8 ± 51.6	204.5 ± 61.6	214.9 ± 72.3
E/e' (lateral) (cm/s)	11.9 ± 5.5	10.8 ± 3.9	11.8 ± 7.3	11.8 ± 5.8
E/e' (medial) (cm/s)	15.4 ± 9.9	12.8 ± 3.1	12.6 ± 4.9	12.1 ± 6.6
RA pressure (estimated) (mmHg)	7.8 ± 4.5	6.2 ± 2.7	10.0 ± 9.8	5.5 ± 1.6
RV systolic pressure (mmHg)	34.8 ± 12.9	34.3 ± 13.3	32.3 ± 9.8	33.4 ± 9.2

§ vs. ≤ 48 hours P < 0.05; † vs. ≥ 2 weeks No improvement P < 0.05

### Left Ventricular Mechanics

#### Global Strains

In comparison to controls, global endocardial and epicardial longitudinal strains were markedly attenuated at 48 hours following PCI (-15 ± 4 vs. -9 ± 4% for endocardium, -12 ± 2 vs. -8 ± 4% for epicardium, P < 0.001 for both). There was an improvement during follow up in 24 (57%) patients (-9 ± 3 vs. -13 ± 5%, P < 0.001 for endocardial longitudinal strains and -8 ± 4 vs. -11 ± 3%, P = 0.003 for epicardial longitudinal strains respectively). Similarly, endocardial circumferential strain was improved at serial follow up (-14 ± 6 vs. -19 ± 7%, P = 0.01), however radial strains and WMSI remained persistently abnormal (Table 4).

**Table 4:** Strain Data

	Post PCI		Follow-up	
	No Improvement	Improvement	No Improvement	Improvement
Global Endocardial Longitudinal Strain (%)	-9.7 ± 5.7	-9.0 ± 3.7	-10.6 ± 3.2	-13.8 ± 4.5*
Global Epicardial Longitudinal Strain (%)	-9.4 ± 5.0	-8.5 ± 4.1	-8.8 ± 3.2	-11.0 ± 3.7*
Endocardial Circumferential Strain (%)	-16.0 ± 8.5	-14.0 ± 6.2	-17.9 ± 7.0	-19.4 ± 7.7§
Epicardial Circumferential Strain (%)	-4.2 ± 2.1	-3.3 ± 1.9	-6.3 ± 2.2§	-5.0 ± 2.1§
Radial Strain (%)	9.5 ± 5.5	8.2 ± 4.1	9.0 ± 3.6	10.7 ± 5.3

*vs. ≤ 48 hours P < 0.001; ^§ ^vs. ≤ 48 hours P < 0.05

#### Variables Associated with the Improvement in LVEF

**Figure 1:**
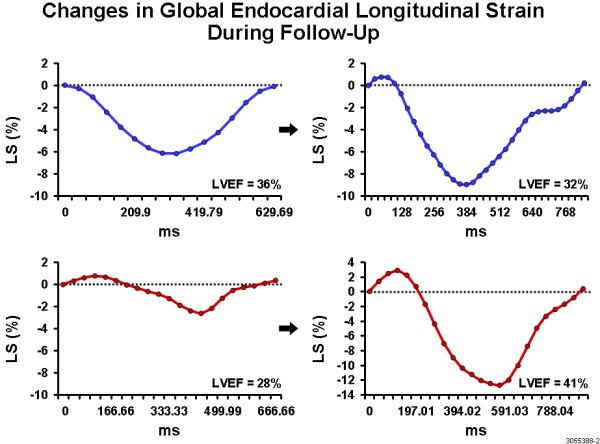
**Panel A and B show attenuated global longitudinal endocardial strain in two patients with anterior wall myocardial infarction**. Note the minimal improvement in longitudinal strain in the first patient (panel C) which has accompanied no improvement in LVEF. In contrast, panel D shows marked improvement in longitudinal strain on follow up in the second patient which is accompanied by significant improvement in LVEF.

**Figure 2:**
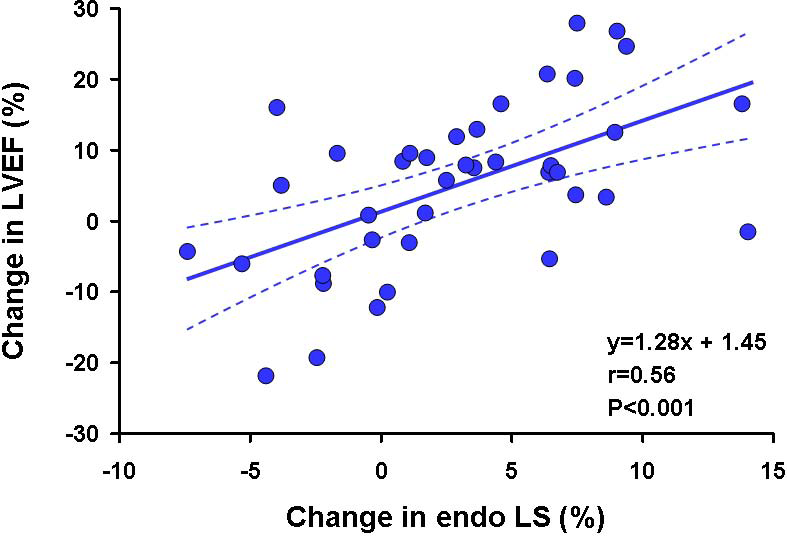
**Correlation between Change of Endocardial Longitudinal Strain and LV Ejection Fraction**.

Among all the clinical variables that were entered into a univariate logistic regression analysis only two were significantly associated with outcome improvement in LVEF at follow up. The change in endocardial longitudinal strain (OR 1.2, 95% CI 1.03 to 1.42, P = 0.01) and the change in epicardial longitudinal strain (OR 1.2, 95% 1.03 to 1.46, P = 0.02) were significantly associated with the improvement in LVEF, independent of the location of STEMI and the presence of underlying multivessel disease (Figures 1, 2) (Table 5).

**Table 5:** Univariate logistic regression Analysis

	Univariate Analysis		
	OR	95% CI	P
Change in Endocardial LS	1.2	1.03 to 1.42	0.01
Change in Epicardial LS	1.2	1.03 to 1.46	0.02
Change in Endocardial CS	1.02	0.96 to 1.10	0.40
Change in Epicardial CS	0.91	0.72 to 1.14	0.42
Change in Radial Strain	0.98	0.92 to 1.04	0.50

LS, Longitudinal Strain; CS, Circumferential Strain

#### Interobserver and Intraobserver Variability

The intraobserver variability for endocardial longitudinal strain, epicardial longitudinal strain, endocardial circumferential strain and epicardial circumferential strain were 10 ± 7%, 8 ± 7%, 11 ± 10%, 25 ± 22% and 24 ± 20% respectively and the kappa coefficients of agreement were 0.57, 0.61, 0.44 and 0.71 respectively. The interobserver variability for the same measurements was -13.6 ± 6.3%, -12.6 ± 7.9%, 16 ± 15%, 26 ± 21% and 28 ± 29% respectively and the kappa coefficients of agreements were 0.42, 0.52, 0.44, and 0.55 respectively. The kappa coefficient intraobserver and interobserver agreement for WMSI was 0.47 and 0.26 respectively.

## Discussion

This study demonstrates that abnormal longitudinal and circumferential strain is frequently present after AMI and improves to a greater degree than abnormal systolic wall motion abnormality in patients with STEMI treated by PCI. Furthermore, improvement of LV longitudinal and circumferential shortening mechanics predicts improvement of global LV function. This new finding contributes to the understanding of how myocardium recovers following acute myocardial ischemia, and is particularly relevant considering that after AMI, the transmural extent of tissue infarction both determines functional recovery and contains prognostic importance. Radial LV mechanics remained persistently abnormal, which may explain the limited ability of WMSI to characterize functional improvements following AMI.

Remodeling is a common phenomenon following acute myocardial infarction (AMI) often accompanied by a decline in left ventricular (LV) ejection fraction (EF). The decline in LVEF is believed to be due in part to loss of initially contracting myocardium and chamber enlargement from progressive post infarction dilatation, resulting in a rise in end systolic volume [13,14]. A number of cellular and molecular changes characterize LV remodeling in this setting including apoptosis [15], fibroblast proliferation [16], and fibrosis [17]. However, the ways in which specific components of LV architecture interact and contribute to improvement of global function after AMI remains incompletely characterized.

One explanation for the lack of radial component improvement observed in this study and the limitation of WMSI in characterizing functional improvements following AMI is that LV wall thickening is not a result of simple shortening of individual myocytes but rather an effect of groups of myocytes shearing across one another. Transmural shearing results from sliding and rearrangement of myofiber sheets along cleavage planes during the cardiac cycle [1-4]. Following MI, the myocardial interstitium is altered by an increase in connective tissue [18]. Furthermore, thickening is a more intricate and complicated process that may not be completely restored after reperfusion [19].

Importantly, there was a trend towards higher prevalence of pre-existing hypertension, hyperlipidemia and diabetes in the group without improvement of global LV function as determined by LV ejection fraction, although the difference did not meet statistical significance. This is not surprising given that both diabetes and hypertension are independent predictors of mortality after STEMI that are incorporated into the TIMI risk score [20]. The duration and effectiveness of treatment of these co-morbidites in this population are unknown, but likely would also have an impact on recovery of LV function due to their roles in LV remodeling and endothelial function.

### Echocardiography in Myocardial Infarction

Echocardiography has several well-established uses in the setting of AMI including the determination of location and extent of ischemia or infarction, detection of complications, and risk stratification of individuals [21]. The wall motion score index (WMSI), a cumulative measure of the burden of abnormally functioning myocardial segments resulting in LV systolic dysfunction, has been useful in determining the prognosis of patients with AMI [22,23]. Morbidity and mortality in patients with AMI is higher in those with a higher WMSI, even in those with relatively preserved ejection fraction [22], highlighting the importance of examining regional segments of LV function. However, WMSI only provides a measurement of primarily radial LV mechanics, which may not accurately represent the complex process occurring within the myocardium after a sustained injury.

### LV Mechanical Function in Myocardial Infarction

More recently, because of their unique ability to detect layer-specific changes in mechanical function, markers of tissue deformation (strain and strain rate) measured by tissue Doppler or speckle tracking are now actively being studied for their potential use as prognostic tools after AMI [24]. Global LV longitudinal strain has been found to correlate with myocardial viability and also predicts recovery of LV function after acute MI [21]. In a recent study, Vartdal and co-authors showed that global LV strain by tissue Doppler imaging was inversely related to infarct size after acute anterior wall MI as determined by gadolinium-enhanced MRI [25]. Another study by Zhang et al. similarly performed on 60 patients with acute MI who underwent strain rate quantification by tissue Doppler imaging as well as contrast-enhanced MRI found that the peak systolic strain rate of transmurally infarcted segments was significantly lower than with normal myocardium or with non-transmurally infarcted segments, thus supporting the ability of strain rate imaging to determine the degree of transmurality of scar tissue following MI [26]. Park et al. recently examined patients with acute MI who underwent reperfusion by either PCI or thrombolysis and found that longitudinal strain by both tissue Doppler and speckle tracking imaging predicted LV dilatation with increased LV end diastolic volume during 18 months of follow-up [27]. Strain also independently predicted death and congestive heart failure in this study [27]. The present study is additive to previous findings in that specifically, longitudinal and circumferential shortening mechanics were the primary predictors of improvement in global LV function rather than radial mechanics and LV wall thickening as determined by the WMSI.

#### Limitations

The present study represents a relatively small number of STEMI patients who received PCI at our institution and had 2-D echocardiography and speckle tracking analysis performed within 48 hours of MI and on average 6 months afterwards. There was variability in the timing of follow-up echocardiography; however the majority of subjects (41 of 42) had follow-up > 2 months after STEMI. The purpose of the study was to do a head to head analysis of WMSI and LV mechanics both of which were performed at the same time point for every subject. Long term clinical outcomes including subsequent major adverse cardiac events and mortality were not measured and will be important to examine for future speckle tracking imaging investigations in this population.

## Conclusions

Abnormal longitudinal and circumferential strain is frequently present after AMI and improves to a greater degree than abnormal systolic wall motion abnormality in patients with STEMI treated by PCI. Improvement in global LV function in STEMI patients treated with PCI occurs primarily by augmentation of LV longitudinal and circumferential shortening mechanics. Left ventricular radial mechanics remain persistently abnormal and may explain the limited ability of WMSI in characterizing functional improvements following PCI.

## Abbreviations

AMI: acute myocardial infarction; EF: ejection fraction; LV: left ventricular; MRI: magnetic resonance imaging; PCI: percutaneous coronary intervention; STEMI: ST-elevation myocardial infarction; WMSI: wall motion scoring index.

## Competing interests

The authors declare that they have no competing interests.

## Authors' contributions

GC and HA carried out LV mechanics analysis, collected data, performed statistical analysis and drafted the manuscript. ME contributed to analysis and interpretation of data and drafting of the manuscript. NB collected data and contributed to study design. SW, SC and FD contributed to the analysis of echocardiography and cardiac catheterization data. PS was responsible for study design, analysis and interpretation of data, manuscript drafting and critical revision. All authors read and approved the final manuscript.

## About the authors

GC is enrolled in PhD program at: University of Messina, Piazza Pugliatti, 1, 98122 Messina, Italy. All authors are affiliated with: Division of Cardiovascular Diseases, Mayo Clinic College of Medicine, Arizona, USA.
